# Subclinical Atrial Fibrillation: To Anticoagulate or Not?

**DOI:** 10.3390/jcm13113236

**Published:** 2024-05-30

**Authors:** Sharath Kommu, Param P. Sharma

**Affiliations:** 1 Department of Hospital Medicine, Marshfield Clinic Health System, Rice Lake, WI 54868, USA; 2 Department of Medicine, UW School of Public Health and Medicine, Madison, WI 53705, USA; 3 Department of Cardiology, Marshfield Clinic Health System, Marshfield, WI 54449, USA; sharma.param@marshfieldclinic.org

**Keywords:** subclinical atrial fibrillation (SCAF), atrial high-rate episodes (AHRE), atrial fibrillation, anticoagulation, DOAC, implantable heart monitor, wearable heart monitor, smart watch

## Abstract

Atrial fibrillation (AF) carries a stroke risk, often necessitating anticoagulation, especially in patients with risk factors. With the advent of implantable and wearable heart monitors, episodes of short bouts of atrial arrhythmias called atrial high-rate episodes (AHREs) or subclinical AF (SCAF) are commonly identified. The necessity of anticoagulation in patients with SCAF is unclear. However, recent randomized controlled trials, the NOAH-AFNET 6 and ARTESIA, have offered insights into this matter. Furthermore, a study-level meta-analysis combining data from both these trials has provided more detailed information. Reviewing the information thus far, we can conclude that DOACs can result in a notable reduction in the risk of ischemic stroke and can potentially decrease the risk of debilitating stroke, albeit with an increased risk of major bleeding. Thus, informed, shared decision-making is essential, weighing the potential benefits of stroke prevention against the risk of major bleeding when considering anticoagulation in this patient population.

## 1. Introduction

Stroke is the second leading cause of death globally, accounting for 11.6% of all deaths in 2019 [1]. Among the numerous causes of stroke, atrial fibrillation (AF) stands out as a significant risk factor, elevating the likelihood of stroke by fivefold [2]. Nonetheless, in 20% to 40% of instances, the root cause of stroke remains elusive [3]. It is speculated that between 10% and 30% of these unexplained strokes could be attributed to undetected AF [4,5,6,7,8,9].

Traditionally, the diagnosis of AF relied on the documentation of the arrhythmia in an electrocardiogram (ECG). However, advancements in technology, including cardiac implanted electronic devices (CIEDs), implantable cardiac monitors (ICMs), and wearable monitors such as adhesive patches with sensors, wristbands, watches, bras, and shirts with embedded leads and sensors, have led to the identification of new categories of atrial arrhythmias. Atrial high-rate episodes (AHREs) are defined as device-detected atrial events, typically tachyarrhythmias, meeting specific atrial high-rate criteria (usually between 175 and 220 bpm) [3]. Subclinical atrial fibrillation (SCAF) is defined as asymptomatic episodes of AF that were previously undetected by standard ECG monitoring but are now identified and confirmed by intracardiac, implantable, or wearable monitors and verified by intracardiac electrogram or a review of the recorded rhythm on the ECG [3]. Scientific societies like the American College of Cardiology (ACC) and the American Heart Association (AHA) have not specified the duration of episodes in defining SCAF or AHREs [3,10]. An AHA scientific statement noted that studies associating SCAF with thromboembolic risk have used variable durations, and the exact threshold for thromboembolic risk remains uncertain [3]. Consequently, the statement does not specify a duration in the definition of SCAF or AHRE. However, it is important to note that AHREs lasting more than 5 min are highly correlated with AF and atrial flutter, whereas shorter episodes often represent other tachyarrhythmias [11]. Additionally, the ASSERT trial, which is one of the major trials to investigate the risk of stroke in subclinical atrial fibrillation, highlighted that SCAF episodes lasting more than 6 min are associated with an increased risk of ischemic stroke or systemic embolism compared to no episodes [12].

AHREs are regarded as biological equivalents to brief episodes of AF or SCAF [13]. However, false positive AHRE recordings may occur with CIEDs due to factors such as far-field R-wave oversensing or lead noise [3]. For instance, in the Asymptomatic Atrial Fibrillation and Stroke Evaluation in Pacemaker Patients and the Atrial Fibrillation Reduction Atrial Pacing Trial (ASSERT), 82.7% of AHREs were confirmed as true atrial tachyarrhythmias or AF, while 17.3% were identified as false positives [14]. Hence, when the device identifies an AHRE, it must be adjudicated to avoid false positives.

Given that AF increases the risk of strokes, the necessity of anticoagulation for stroke prevention in cases of short bouts of SCAFs/AHREs has been uncertain till recently, particularly due to the lack of large-scale randomized trials investigating this question. However, recent randomized-controlled, blinded trials, namely the Non-Vitamin K Antagonist Oral Anticoagulants in Patients with Atrial High-Rate Episodes (NOAH-AFNET 6) [15] and Apixaban for the Reduction of Thrombo-Embolism in Patients with Device-Detected Subclinical Atrial Fibrillation (ARTESIA) [16] trials, have provided more clarity on this matter by investigating the benefits and risks associated with anticoagulation in these patient cohorts. The NOAH-AFNET 6 trial compared edoxaban to a placebo, while the ARTESIA trial compared apixaban to aspirin. Notably, 53.9% of patients in the placebo group of the NOAH-AFNET 6 trial were also on aspirin, drawing a closer comparison to the control (aspirin) group of the ARTESIA trial [15]. NOAH-AFNET 6 enrolled patients with AHREs (considered biological equivalents of SCAF), while ARTESIA included patients with SCAF. Previously, various studies have used different cutoffs to define the threshold time for AHREs and SCAFs. However, these two trials have used a similar cut of 6 min or more. These trials examined outcomes such as the risk of ischemic stroke, major bleeding, cardiovascular death, and all-cause mortality.

## 2. Results of the Studies

The characteristics of NOAH-AFNET 6 and ARTESIA trials are shown in Table 1. Both studies are double-blind, double-dummy randomized trials. The median duration of follow-up in the NOAH-AFNET 6 trial was 21 months, while the mean follow-up duration for the ARTESIA trial was 3.5 years. While the NOAH-AFNET 6 trial included patients with at least one risk factor for stroke, the ARTESIA trial included patients with a CHA_2_DS_2_-VASc score (a scoring system utilized to forecast the likelihood of ischemic stroke among patients with AF, ranging from 0 to 9, where higher scores signify an increased risk of stroke) of 3 or more. The median CHA_2_DS_2_-VASc score in the NOAH-AFNET 6 trial was 4, and the mean score was 3.9±1.1 in the ARTESIA trial. In the ARTESIA trial, patients with SCAF were included if the duration was equal to or exceeded 6 min but was less than 24 h. Conversely, the NOAH-AFNET 6 trial enrolled patients with AHREs with a minimum duration of 6 min, while the upper limit was not specified. However, the mean duration (with interquartile range) in both treatment and control groups in the NOAH-AFNET 6 trial was 2.8 h (0.8–9.2) and 2.8 h (0.7–9.5), respectively [15], indicating that episodes did not exceed 24 h in duration.

The NOAH-AFNET 6 trial was halted prematurely due to safety concerns, with only 184 out of the intended 220 primary outcomes occurring, leading to insufficient power for the study [15]. Similarly, in the ARTESIA trial, only approximately two-thirds of the planned primary events transpired, prompting an early conclusion due to lower-than-expected event rates [16]. A study-level meta-analysis was performed by pooling data from these trials, which addressed the limitation related to the insufficient power of these studies and provided a more nuanced understanding of the benefits and risks of anticoagulation in SCAF [17].

Both randomized trials had different primary efficacy outcomes. The primary efficacy outcome in the NOAH AFNET 6 trial (the first occurrence of a composite of cardiovascular death, stroke, or systemic embolism) did not differ between the edoxaban and placebo groups (HR 0.81, 95% CI 0.60 to 1.08) [15]; similarly, the risk of stroke or systemic embolism did not differ between the two groups in this trial (HR 0.65, 95% CI 0.39 to 1.07) [15]. In contrast, the ARTESIA trial found a significant difference in stroke or systemic embolism between the apixaban and aspirin groups (HR 0.63, 95% CI 0.45 to 0.88) [16]. A meta-analysis combining both trials showed a lower risk of stroke or systemic embolism in the DOAC group compared to the aspirin/placebo group (RR 0.65, 95% CI 0.49 to 0.86) [17].

The risk of ischemic stroke did not differ in the edoxaban group compared to the placebo group in the NOAH-AFNET 6 trial (HR 0.79, 95% CI 0.45 to 1.39) [15]. However, in the ARTESIA trial, this risk was lower in the apixaban group compared to the aspirin group (HR 0.62, 95% CI 0.43 to 0.91) [16]. The meta-analysis showed a lower risk of ischemic stroke in the DOAC group compared to the aspirin/placebo group (RR 0.68, 95% CI 0.50 to 0.92) [17].

The risk of major bleeding was higher in the DOAC group compared to the control group in both trials. In the NOAH-AFNET 6 trial, the HR was 2.10 (95% CI 1.30 to 3.38) [15], while in the ARTESIA trial, it was 1.36 (95% CI 1.01 to 1.82) for this outcome [16]. The meta-analysis confirmed a higher risk of major bleeding in the DOAC group compared to the aspirin/placebo group (RR 1.62, 95% CI 1.05 to 2.5) [17]. However, the risk of fatal bleeding did not differ between the groups (RR 0.79, 95% CI 0.37 to 1.69) in the meta-analysis [17].

The risk of cardiovascular death in patients with SCAF did not differ in the NOAH-AFNET 6 (edoxaban vs. placebo, HR 0.90, 95% CI 0.62 to 1.31) [15] and the ARTESIA (apixaban vs. aspirin, HR 0.96, 95% CI 0.73 to 1.25) [16] trials. The pooled analysis confirmed a similar risk of cardiovascular death between the DOAC and aspirin/placebo groups (RR 0.95, 95% CI 0.76 to 1.17) [17]. Similarly, the risk of all-cause mortality was not different in the NOAH-AFNET 6 (HR 1.16, 95% CI 0.88 to 1.53) [15] and the ARTESIA (HR 1.04, 95% CI 0.90 to 1.21) [16] trials. The combined analysis also showed no difference in all-cause mortality (RR 1.08, 95% CI 0.96 to 1.21), indicating a neutral effect of DOACs on this outcome [17].

## 3. Discussion

One of the earlier randomized trials that investigated device-detected AF and anticoagulation was the implantable loop recorder detection of atrial fibrillation to prevent stroke (The LOOP Study) [18]. It demonstrated that in patients with risk factors for stroke, implantable loop recorders (ILRs) increased the detection of AF episodes lasting over 6 min (SCAF), leading to increased initiation of anticoagulation therapy [18]. However, there was no observable reduction in the occurrence of stroke or systemic embolism within the ILR-implanted group when compared to the control group. However, the Kaplan–Meyer curves portraying the incidence of stroke or systemic embolism began to diverge around the 2–3-year mark, showing a trend favoring the ILR group [18]. It is important to note that in this study, 91% of those detected with AF in the ILR group and 86.5% in the control group received anticoagulation [18]. While this study’s findings provide valuable insights, it did not directly address the question of anticoagulation versus no anticoagulation in patients specifically diagnosed with device-detected SCAF. The NOAH-AFNET 6 and the ARTESIA trials and the study-level meta-analysis of these trials addressed this knowledge gap.

Evidence shows that in comparison to clinical AF, SCAF poses a reduced risk of stroke. For instance, the TRENDS cohort, which monitored patients with prolonged AHRE episodes, reported an annual stroke rate ranging from 1.1% to 2.2% [19]. However, such a risk in clinical non-valvular AF averages around 5% per year [20]. From the results of the NOAH-AFNET 6, ARTESIA, and their study level meta-analysis, there is an indication that the risk of stroke in patients with SCAF (though lower than in patients with clinical AF) can be further mitigated with anticoagulation therapy, albeit with an increased risk of major bleeding [15,16,17]. Consequently, making recommendations regarding anticoagulation in patients with SCAF becomes challenging. Along similar lines, the 2023 American College of Cardiology/American Heart Association Joint Committee on Clinical Practice Guidelines for AF offers a weaker recommendation (2B) for anticoagulation in patients with short-duration AF and a high risk of stroke, defined by a CHA_2_DS_2_-VASc score of ≥3 [10].

Both the trials, as well as the meta-analysis, showed an increased risk of major bleeding in the DOAC group compared to the aspirin/placebo group. Based on the size and event numbers in both randomized trials, we calculated that in patients with SCAF, the use of DOACs can result in one fewer stroke per 210 DOAC recipients per year and one excess major bleeding event per 149 DOAC recipients per year, indicating a slightly higher risk of major bleeding, compared to ischemic strokes prevented. However, it is noteworthy that the number of fatal bleeding events was low in the DOAC and aspirin/placebo groups and did not reach statistical significance, as demonstrated in the meta-analysis [17].

An argument can be made that bleeding can often be managed by measures such as discontinuing anticoagulation, administering blood transfusions, and using reversal agents in severe cases, while stroke can be debilitating and can leave lasting effects. The ARTESIA trial demonstrated that the risk of debilitating stroke (modified Rankin scale of 3 to 6) was higher in the control (aspirin) group (0.53% per patient-year) compared to the apixaban group (0.27% per patient-year) with a hazard ratio of 0.51 (95% CI 0.29 to 0.88) [16], indicating a significant benefit with apixaban in preventing debilitating stroke in patients with SCAF. Hence, we may favor the use of anticoagulation in patients with SCAF, particularly in individuals with a high risk of stroke, who can promptly monitor bleeding and report any concerns. A modifiable bleeding risk should be corrected before anticoagulation is considered. In patients who have a continued high risk of bleeding, left atrial appendage occlusion can be considered to decrease the risk of stroke, particularly in those with multiple risk factors; however, the utility of this procedure in SCAF has to be clearly defined. Nonetheless, physicians must exercise caution in recommending for or against anticoagulation in patients with SCAF, and informed, shared decision-making with the patient is paramount.

We should also consider the duration of SCAF in our decision-making. The ASSERT trial, conducted by Healey et al., revealed that longer durations of subclinical atrial tachyarrhythmias are associated with a higher risk of stroke [12]. The study indicated that for episodes lasting ≤0.86 h, the annual rate of stroke or systemic embolism was 1.23 (95% CI, 0.15 to 4.46) [12]. In the duration range of 0.87 to 3.63 h, the risk was 0 (95% CI, 0 to 2.08), while for durations between 3.64 and 17.72 h, it was 1.18 (95% CI, 0.14 to 4.28) [12]. Notably, for episodes lasting >17.72 h, the risk significantly increased to 4.89 (95% CI, 1.96 to 10.07) [12]. Although the study was underpowered for this specific analysis, it provided valuable insights into the escalating risk of stroke or systemic embolism associated with prolonged SCAF durations.

**Figure 1:**
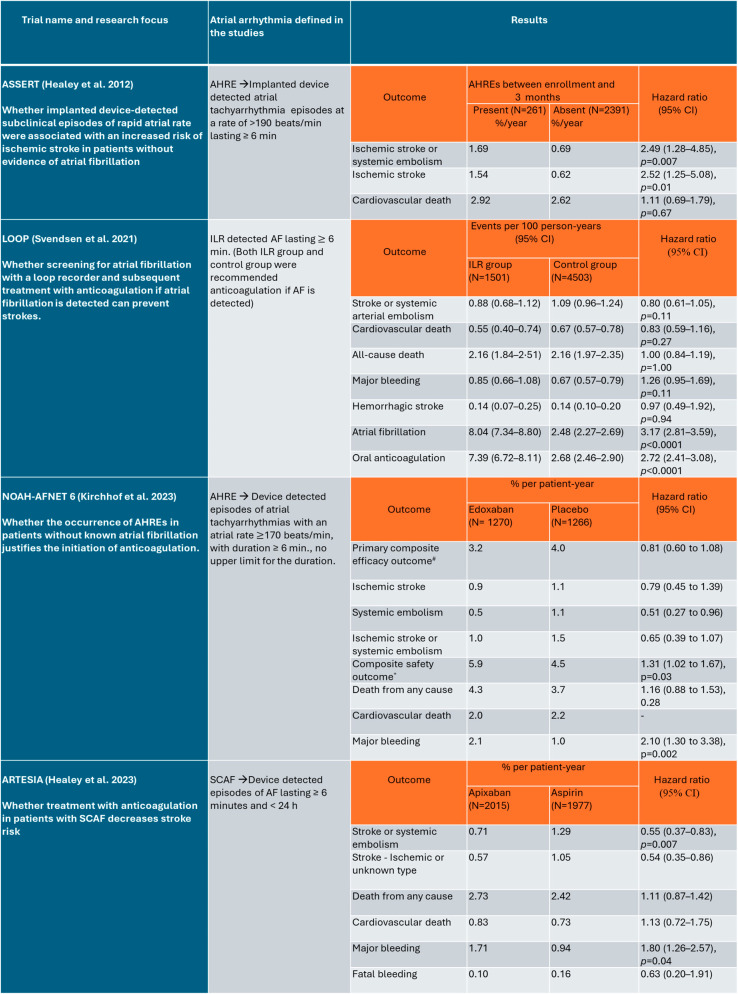
Major studies on SCAF/AHRE that included large patient populations (over 2500 participants per study), which were utilized for this analysis. SCAF—subclinical atrial fibrillation, AHRE—atrial high-rate episode, ILR—implantable loop recorder, AF—atrial fibrillation, N—number of patients, CI—confidence interval, #—first occurrence of a composite of cardiovascular death, stroke or systemic embolism, *—a composite of death from any cause or major bleeding [12,15,16,18].

**Figure 2:**
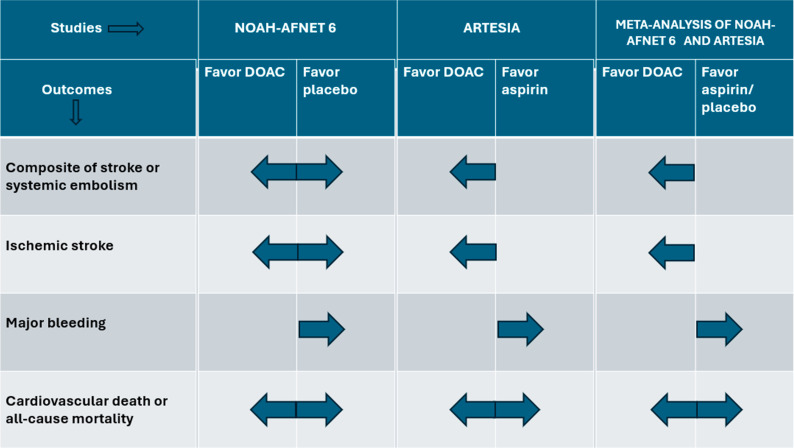
Summary figure showing the major efficacy and safety outcomes with anticoagulation in patients having sub-clinical atrial fibrillation with stroke risk factor(s) in the NOAH-AFNET 6 trial, ARTESIA trial, and their study-level meta-analysis. NOAH-AFNET 6—Non-Vitamin K Antagonist Oral Anticoagulants in Patients with Atrial High-Rate Episodes Trial [15], ARTESIA—Apixaban for the Reduction of Thrombo-Embolism in Patients with Device-Detected Subclinical Atrial Fibrillation Trial [16], DOAC—direct oral anticoagulant. Dark blue arrow indicates the direction of effect.

Several studies give insight into the realm of device-detected SCAF. Figure 1, below, highlights the large-scale studies on SCAF/AHRE and their main results, and Figure 2 summarizes the benefits and risks of anticoagulation in patients with SCAF.

The CHA_2_DS_2_-VASc scoring system is commonly employed to assess stroke risk in patients with AF. There needs to be clarity about the scoring system that can be used in predicting the risk of stroke in patients with SCAF. Individuals with SCAF typically exhibit a lower stroke risk compared to those with clinically diagnosed AF, potentially resulting in an overestimation of risk when using the same scoring system in individuals with SCAF. They may benefit from higher threshold values for scoring or a modified scoring system to assess stroke risk, and this may facilitate clinical decision-making more accurately. Currently, a duration threshold is not included in the definition of SCAF. However, based on the thresholds used in the two major trials discussed here (NOAH-AFNET 6 and ARTESIA), it is possible that a period of 6 min to 24 h may be added in future definitions of SCAF.

It is also important to note that while only a minority of patients with SCAF (approximately 13–16% over 2.5 years) are estimated to progress to clinical AF [12,21], it remains crucial to monitor these individuals closely. This vigilance is essential as patients with established AF face a higher risk of stroke; however, they have better-defined guidelines regarding anticoagulation therapy for stroke prevention.

The outcomes of cardiovascular death and all-cause mortality in both the trials (NOAH AFNET 6 and ARTESIA) and their study-level meta-analysis were almost similar, with no statistically significant difference between the treatment and control arms. Large-scale randomized trials over longer durations may provide further insight into whether there will be any benefits in these outcomes over the long run.

## 4. Conclusions

From the information we have thus far, we can conclude that in individuals with SCAF (duration of ≥6 min and <24 h) with risk factor(s) for stroke, anticoagulation can significantly reduce the risk of ischemic stroke, albeit with an increased risk of major bleeding. Given these findings, when considering anticoagulation for stroke prevention in patients with SCAF, shared decision-making becomes paramount. It is essential to carefully weigh the benefits of stroke prevention against the potential risk of major bleeding in these cases. Continued large-scale studies on anticoagulation in patients with SCAF or AHREs are essential for a better understanding of the benefits and risks of anticoagulation over the long term.

## Figures and Tables

**Table 1:** The table showing the characteristics of two randomized-controlled trials investigating the advantages and risks of anticoagulation in subclinical atrial fibrillation patients, comparing outcomes between direct oral anticoagulants (DOACs) and a control group.

Trial Name	Study Design and Location	Study Duration	Test Drug (*n*)/Control (N)	Mean Age	CHA_2_DS_2_-VASc Score	Arrhythmia	Stroke or Systemic Embolism	Ischemic Stroke HR (95% CI)	Major Bleeding HR (95% CI)	Cardiovascular Death HR (95% CI)	All-Cause Mortality HR (95% CI)
NOAH-AFNET6 trial. Kirchhof et al. (2023) [15]	Investigator-initiated, DB, double-dummy, RCT. Multicenter.	5 years	Edoxaban (1270)/Placebo (1266)	77.5 ± 6.7	4 ± 1 (Median)	AHRE *	0.65 (0.39 to 1.07)	0.79 (0.45 to 1.39)	2.10 (1.30 to 3.38)	0.90 (CI, 0.62 to 1.31)	1.16 (0.88 to 1.53)
ARTESIA trial. Healey et al. (2023) [16]	DB, double-dummy randomized trial. Multicenter	6 years	Apixaban (2015)/Aspirin (1997)	76.8 ± 7.6	3.9 ± 1.1 (Mean)	SCAF #	0.63 (0.45 to 0.88)	0.62 (0.43 to 0.91)	1.36 (1.01 to 1.82)	0.96 (0.73 to 1.25)	1.04 (0.90 to 1.21)

NOAH-AFNET 6—Non-Vitamin K Antagonist Oral Anticoagulants in Patients with Atrial High-Rate Episodes Trial [15], ARTESIA—Apixaban for the Reduction of Thrombo-Embolism in Patients with Device-Detected Subclinical Atrial Fibrillation Trial [16], *n*—number of patients, HR—hazard ratio, CI—confidence interval, RCT—randomized controlled trial, DB—double-blind, AHRE—atrial high-rate episode, SCAF—subclinical atrial fibrillation. *—AHREs included in this study are the atrial tachyarrhythmia episodes detected at least 2 months after implantation of the device, with an atrial rate of ≥170 beats per minute and had to last ≥6 min; no upper limit for the duration. #—SCAFs included in this study are the device-detected AF episodes, with at least one episode lasting ≥6 min but not longer than 24 h.

## Data Availability

Not applicable.
